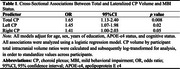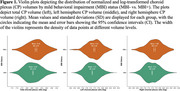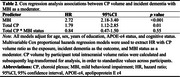# Analyzing choroid plexus volume in association with mild behavioral impairment and progression to dementia

**DOI:** 10.1002/alz70856_097751

**Published:** 2025-12-24

**Authors:** Sabika Azhar, Dylan X. Guan, Maryam Ghahremani, Zahinoor Ismail

**Affiliations:** ^1^ University of Calgary, Calgary, AB, Canada; ^2^ Hotchkiss Brain Institute, University of Calgary, Calgary, AB, Canada

## Abstract

**Background:**

Mild behavioral impairment (MBI) improves early Alzheimer's disease (AD) detection by leveraging risk associated with later‐life emergent and persistent neuropsychiatric symptoms. The choroid plexus (CP), a capillary network forming the blood‐CSF barrier and producing cerebrospinal fluid (CSF), supports brain homeostasis and clearance of toxic molecules disrupted in AD. CP volume expansion precedes dementia onset, correlating inversely with cognitive performance; links with MBI remain unexplored. Here, we investigated CP volume differences in dementia‐free individuals with and without MBI, and evaluated whether CP volume predicts progression to dementia, moderated by MBI.

**Method:**

Data were obtained from the National Alzheimer's Coordinating Center. Behavioral status was operationalized using a published algorithm applied to the Neuropsychiatric Inventory Questionnaire. T1‐weighted magnetic resonance imaging scans were processed with FreeSurfer and segmented using a Gaussian mixture model segmentation algorithm to estimate bilateral CP volumes. Logistic regressions modeled the association between CP volume (exposure) and MBI status (outcome), adjusting for age, sex, education, cognitive status, and apolipoprotein E ɛ4 status. Cox proportional hazards regression modeled the association between CP volume (exposure) and incident dementia (outcome), with MBI status as a moderator, adjusting for the same covariates.

**Result:**

Among 2111 participants (43.0% female, 30.2% with Mild Cognitive Impairment, mean age 71.3±8.8 years), greater CP volumes were associated with 65% higher odds of MBI (95%CI[1.13‐2.40], *p* = 0.008) (Table 1). Findings for left and right CP volumes were similar, with left CP volumes associated with 45% higher odds of MBI (95%CI[1.07‐1.98], *p* = 0.02) and right CP volumes with 41% higher odds (95%CI[1.00‐2.03], *p* = 0.05), indicating no notable hemispheric differences for associations with MBI. MBI+ status had a higher hazard ratio (HR) for incident dementia (HR=2.72), as did higher CP volume (HR=1.79). However, MBI+ status did not moderate the association between CP volume and incident dementia (*p* = 0.55) (Table 2).

**Conclusion:**

MBI+ participants had higher CP volumes, and both MBI+ status and CP volumes independently predicted incident dementia. While MBI did not moderate the CP‐dementia association, it might serve as a mediator, warranting further research. These findings support MBI as a clinical tool for identifying dementia risk, and highlight CP volume as a potential neuroimaging biomarker.